# National asthma observational survey of severe asthmatics in Israel: the no-air study

**DOI:** 10.1186/1710-1492-8-8

**Published:** 2012-06-01

**Authors:** Gabriel Izbicki, Anna Grosman, Zeev Weiler, Tiberiu Shulimzon, Uri Laxer, Gershon Fink

**Affiliations:** 1 Pulmonary Institute, Shaare Zedek Medical Center, affiliated with the Hebrew University-Hadassah School of Medicine, Jerusalem, 91031, Israel; 2 Pulmonary consultant at Maccabi and Meuhedet Sick Funds, Jerusalem, 91031, Israel; 3 Pulmonary Institute, Barzilai Medical Center, Ashkelon, 78306, Israel; 4 Pulmonary Institute, Sheba Medical Center, Tel Hashomer, 52621, Israel; 5 Pulmonary Institute, Hadassah Medical Center, Jerusalem, 91120, Israel; 6 Pulmonary Institute, Kaplan Medical Center, Rehovot, 76100, Israel; 7 Director Pulmonary Institute, Shaare Zedek Medical Center, Affiliated with the Hebrew University-Hadassah School of Medicine, P.O. Box 3235, Jerusalem, 91031, Israel

**Keywords:** Asthma, GINA, Survey, Severe, Disease-control, Asthma-control, Questionnaire, Medication, Compliance, Guidelines

## Abstract

**Background:**

Asthma is considered a global public health issue requiring a significant medical expenditure as a result of its high prevalence and the low rate of disease control.

**Objective:**

This is the first nationwide survey of severe asthma patients carried out in Israel. In this study we aimed to assess health resources utilization, compliance with treatment and disease-control in a subgroup of patients with severe asthma in Israel.

**Material and method:**

One hundred and twenty-three patients with a diagnosis of asthma for more then one year, as well as a hospitalization during the last 12 months due to asthma exacerbation or maintenance systemic steroids therapy, were included in this non-interventional observational study.

**Results:**

Asthma was uncontrolled in 43.9%, partly controlled in 50.4% and well controlled in only 5.7%. The majority of the patients (83%) were compliant with drug treatment.

**Conclusion:**

The fact that 83% of the asthma patients included in this study were compliant with their asthma therapy was not manifested in asthma control. Therefore concrete tools are required for achieving and maintaining asthma control, especially in the treatment of the most severe asthmatic patients.

## Introduction

Asthma represents a major public health concern, with increasing incidence and prevalence worldwide [1]. Asthma affects over 300 million people all over the world and is one of the most common chronic diseases [2,3]. Estimates of the prevalence of asthma range from 7% in France and Germany, to 11% in the USA and 15-18% in the United Kingdom [2]. Approximately 20% of these patients have severe asthma, of which 20% are inadequately controlled [4]), and these patients are at particularly high risk of exacerbation, hospitalization and demise often suffering from a radically impaired quality of life. The medical expenditure related to asthma deriving from work absences, missed school days and increasing healthcare costs has raised this disease to high priority in terms of finding new therapeutic as well as improved compliance modalities.

Various studies indicate that the burden of illness is not necessarily associated with asthma itself but with the lack of asthma control [5]. The unfavorable effects of inadequate asthma control range from increased risk of exacerbation and emergency visits, to hospitalization and death [6,7].

The aim of this survey was to assess health resources utilization, compliance with treatment and disease-control in a subgroup of patients with severe asthma in Israel.

## Material and methods

### Study population and procedures

This is a non-interventional observational study based on a single patient visit conducted in 12 Israeli centers by pulmonologists, all members of the Israeli Pulmonary Society. The patients completed Hebrew-translated standard, international used questionnaires regarding asthma control, therapy compliance and quality of life, and the pulmonologists completed questionnaires with regard to severity, disease-control, comorbidities and treatment. More specifically, questionnaires were divided into five parts: 1) demographics 2) health resource utilization 3) compliance to treatment based on the standard Asthma Compliance Test [8], 4) level of asthma-control based on the standard Asthma Control Test [9]) and 5) asthma history and treatment. The first 3 parts were completed by the patients and the last 2 parts by the physicians. Patient's questionnaires were self-administered and completed in the out-patient clinic waiting area while physicians completed their questionnaires just after the patient's visit.

Patients above the age of 18 were included in this study after signing informed consent. Inclusion criteria were a diagnosis of asthma (based on the ATS and GINA guidelines) for more then one year together with either a hospitalization during the last 12 months due to asthma exacerbation, or maintenance systemic steroids therapy for at least 50% of the year. Total IgE level from up to 12 months before visit 1 until one month after visit 1 was obtained. All patients fulfilling inclusion criteria were asked to participate in this survey, during a 12 month period of time. Asthmatic patients who were hospitalized for reasons other than asthma, or patients with a diagnosis of COPD, were excluded from this survey. Asthma-control was assessed for every patient according to the Global Initiative for Asthma (GINA) update of 2006 [10,11].

### Ethical considerations

This observational survey was performed according to the principles of Good Clinical Practice, the Helsinki Declaration, and national laws and regulations for clinical studies. This survey received extraordinary approval from the Israeli Ministry of Health which authorized the participation of all affiliated pulmonologists in this study based upon the approval of the Independent Ethics Committee of Kaplan Medical Center (Rehovot) and receipt of the patients’ written informed consent.

## Results

### Demographics, socio-economic and medical history

Results are presented in Table 1. One hundred and twenty-three patients were included in this survey during 12 months. One hundred and twelve (91.1%) were Jews. Seventy one percent of the participants were un-employed (n = 87); 84.8% of the un-employed were either retired (44% of them) or unable to work for health reasons (46% of them). Twenty-four percent had pets at home, of which 72.4% were dogs.

**Table 1:** Demographics – Socio-economic and medical history

Total Number of recruited patients	123
*Age median (range)	52 (19-87)
Married	80 (65%)
Completed high school	76 (62%)
Employed	36 (29%)
Living in urban areas	97 (79%)
Hypertension	38 (31%)
Diabetes Mellitus	22 (18%)

All the results are presented as: n (% of the total).

*Age is expressed as median (range).

### Asthma-related information

Asthma-related information is presented in Table 2. IgE levels were available in 101 patients, the median level was 200 Units/ml. Only 3 patients were current smokers (2.4%). The median number of exacerbations, hospitalizations in the intensive care unit and intubation episodes was also recorded (Table 3). According to the Global Initiative for Asthma (GINA) classification [11], asthma was partly controlled in 50.4% of the patients, uncontrolled in 43.9%, and well controlled in only 5.7%.

**Table 2:** Asthma related information

Diagnosis of asthma below the age of 40	93 (75.6%)
Allergic rhinitis	68 (55%)
Food or drug allergy	24 (19.5%)
Atopic dermatitis	16 (13%)
Never smoked	98 (80%)
Ex-smokers	22 (17.9%)
*FEV1	57%
*PEF	53%
#Positive Skin test/RAST	32/46 (69.5%)

**Table 3:** Exacerbations, Hospitalizations & Intubation

Median number of exacerbations within the last 12 month (0-25)	4
Median number of exacerbations over the past 5 years (0-100)	5/year
History of hospitalization in ICU and intubation	17.0%

### Treatment

Ninety percent of the patients were treated with short-acting beta-2-agonists (mostly salbutamol/albuterol), 19.2% with long-acting beta-2-agonists (LABA) (formoterol preferred among other LABA), 15% inhaled corticosteroids (budesonide preferred), and 83.7% inhaled combination therapy (salmeterol/fluticasone preferred). Other drugs included montelukast (n = 37, 30%), theophylline (n = 29, 23.6%), and omalizumab (n = 26, 21.1%). No patient received LABA alone. The majority (68%) of the patients receiving LABA did so on-top of inhaled combination therapy while the rest (32%) were treated with separate LABA and inhaled corticosteroids. Amongst patients treated with inhaled combination therapy 87% received the maximal dose (salmeterol/fluticasone 500 mcg two times per day or formoterol/budesonide 320mcg two times per day and only 13% received a smaller dose (salmeterol/fluticasone 250 mcg two times per day or formoterol/budesonide 160mcg two times per day. Forty-three percent received corticosteroids systemically (prednisone preferred) as a maintenance therapy. The most common treatment-associated adverse events included osteoporosis, obesity, and hypertension.

### Quality of life and compliance

Visits to the emergency room, number of hospitalizations and the average hospital-stay is shown in Table 4. Information about compliance and asthma-control is shown in Table 5. More than half of the patients refrained from continuous steroid use out of fear of side effects. The majority of the patients (83%) declared full compliance with their prescribed medications and 92.6% were fully aware of the need for continuous preventive treatment. Sixty-five percent of the patients declared that asthma restricted their activities frequently or permanently. Forty-nine percent of all 123 patients stated that their asthma was not at all, or only partially, controlled, whereas only 8.3% thought it was well controlled. Forty-three percent of the patients did not answer this question. In the subgroup of patients treated with systemic corticosteroids (n = 53) and also amongst patients which received omalizumab (n = 26) there was a trend to better asthma control which did not however reach statistical significance.

**Table 4:** Quality of Life: results expressed as median (range) or percentage of total

Visits to the ER	2 (0-24)
Hospitalizations within last 12 months	1 (0-20)
Hospitalizations in last 5 years	5 (0-60)
Duration of hospital stay	4 (1-25)
Working days lost due to asthma	15 (0-365)
Asthma attacks daily	65.0%
Woke up 4 or more times a night per week	48.8%

**Table 5:** Compliance and asthma-control

Use steroids daily	43.0%
Afraid/very afraid of side effects	51.2%
Stated they were always compliant with treatment	83.0%
Aware of the need for continuous treatment	92.6%
Thought lack of compliance would have a negative impact on their disease	95.0%
Thought treatment with tablets was more efficient than inhalation.	39.0%
Denied the last statement	17.4%
Stated asthma was partially or not-at-all controlled	49.0%
Stated asthma was well controlled	8.3%

## Discussion

This is the first nationwide survey undertaken in Israel concerning severe asthma patients. In this study we found that the majority of the patients (83%) were compliant with drug treatment. However this high self-declared compliance did not translate into asthma control. According to 2006 GINA assessment, 94.3% of patients had partially-controlled or uncontrolled asthma in this study. The median number of exacerbations within the last year was four. The results of this survey are based on questionnaires filled in by patients and physicians, and differ somewhat from the results of the telephone survey performed by Chapman et al. [7] in Canada, although both surveys confirmed the high prevalence of uncontrolled asthma. Forty-nine percent of all 123 patients stated that their asthma was not at all or only partially controlled. Forty-three percent of the patients did not answer this question, but taking into account other answers (i.e., 65% of patients stated they had daily asthma attacks) we can evaluate that the percentage of patients with partially or non-controlled asthma is very high. This assumption is also based on the fact that only 8.3% of the patients thought that their asthma was fully controlled. The pulmonologists found that only 5.7% of the patients had well-controlled asthma, which is quite consistent with the patients’ self-evaluation of well-controlled disease. In the Reality of Asthma Control (TRAC) study [12] 53% of adults who reported having asthma were uncontrolled. In the INSPIRE study [13] 51% of the patients had uncontrolled asthma, as assessed by the Asthma Control Questionnaire. Although study populations and methodology are very different in these two studies the percentage of uncontrolled asthma is quite similar. In 2009 Demoly et al. published a cross-sectional study of 37,476 adults in France, Germany, Italy, Spain and the UK in which 50.4% of asthma patients reported having partially-controlled disease [2]. Severe persistent asthma causes a substantial morbidity and mortality burden and is frequently inadequately controlled despite intensive guideline-based therapy. The high percentage of patients with asthma under little or no control in our study is partially due to the extremely restrictive inclusion criteria of our survey. Nevertheless, according to GINA guidelines, asthma control can be achieved and maintained amongst the majority of asthmatics. In our study, although 83.7% of the patients used combination therapy (inhaled steroids and LABA) only 30% used montelukast and 21% omalizumab, despite the severity and poor control-level of their disease. Although there was a trend amongst better asthma control in the subgroup of patients treated with corticosteroids systemically or Omalizumab, this difference did not reached statistically significance as compared with the total population. As many as 83% of the patients reported constant compliance with their prescribed treatment. We had no possibility to reconcile the patient's reports concerning the high compliance to treatment, with prescriptions refills or other more objective measure. There are more objective methods to assess compliance with treatment but as this was a non-interventional, observational survey, we were limited to the use of questionnaires only. Although this information is based on patients report only, authors believe that there is room for improvement of current treatment regimens in the subset of patients presented in this study.

The limitations of this survey are firstly the small size of the study group. The relatively small size of the group can be explained by the restrictive inclusion criteria. It is not a general survey of asthma control in Israel but a survey of a severe asthmatic population. Moreover, as participation to this national survey was on a volunteer basis, not all the hospitals and pulmonologists, took an active part in this survey. Nevertheless we do think that this is a representative sample of the sub-population of patients with severe asthma. It should also be stressed that since there is no uniform definition of severe asthma, the results of this study are limited to the recruited population and not to all severe asthmatics. Secondly, we did not analyze the association of asthma control with other diseases, health attitudes (obesity, smoking,) or treatments. The third limitation is the use of the questionnaire to assess compliance with asthma medication. The patients' questionnaires were self-administered and completed. There are more objective methods to assess compliance but as this was a non-interventional survey we used validated questionnaires. Finally it might be argued that surveying patients in hospital facilities would be biased towards including mostly uncontrolled asthma patients since such patients are more likely to require care in a hospital setting.

## Conclusions

In this survey, asthma impaired quality of life and was a major cause of hospitalizations, emergency room visits and absence from work/school. Although most patients surveyed in this study (83%) declared full treatment compliance, this overwhelming number did not translate into satisfactory disease control, as only 5.7 - 8.3% of the patients (physicians’ versus patients’ evaluation), claimed well controlled asthma. Several studies, including this survey, show that the illness burden is not only associated with the severity of the disease but mostly with the lack of asthma control. More concerted efforts in achieving and maintaining control are needed. Other treatment options for severe asthmatic patients should be considered since most of the severe patients were not well controlled.

## Appendix 1

List of centers participating in this trial who recruited more then 5 patients, Name of PI (and number of patients they recruited).

• Kaplan Medical Center, Rehovot, Dr. G. Fink, (30).

• Shaare Zedek Medical Center, Jerusalem, Dr. G. Izbicki (15)

• Barzilai Medical Center, Ashkelon, Dr. Z. Weiler (11)

• Macabi and Meuhedet Sick funds, Jerusalem, Dr. A. Grossman (11)

• Sheba Medical Center, Tel-Hashomer, Prof. Y. Ben-Dov (10)

• Hadassah Medical Center, Jerusalem, Prof. R. Breuer (10)

• Assaf Harofeh Medical Center, Zrifin, Dr. D. Stav (8)

• Hillel Yaffe Medical Center, Hadera, Dr. M. Bekerman (7)

• Haemek Medical Center, Afula, Dr. M. Yunis (7)

• Poria Medical Center, Tiberias, Dr. C. Sismolo (5)

• Three other centers recruited a total of nine patients together.

## Competing interest

The authors report no competing interests. The authors alone are responsible for the content and writing of this paper.

## Authors’ contribution

GI and GF contributed to Conception and design of the study, acquisition, analysis and interpretation of data, drafting the manuscript and final approval. They were also involved in acquisition of funding, collecting data and general supervision. AG, ZW, TS and UL were involved in Acquisition of data, analysis and interpretation of data, significant revision of the manuscript and final approval. All authors read and approved the final manuscript.

For the list of all centers participating in this survey and PI of each center see Appendix 1.

The abstract was presented at the annual scientific meeting of the Israel Pulmonary Society in 2011.
